# Evolution of XDR *Pseudomonas aeruginosa* ST463 strains with two plasmids harboring multiple antimicrobial resistance genes

**DOI:** 10.1128/aac.01697-24

**Published:** 2025-07-23

**Authors:** Yinfei Fang, Meijun Song, Yisha Zhang, Zhaoxia Wu, Xiaobing Li, Yuxiang Guo, Nanfei Wang, Xiaoting Hua, Yunsong Yu

**Affiliations:** 1 Department of Clinical Laboratory, Affiliated Jinhua Hospital, Zhejiang University School of Medicine 26441 , Jinhua, Zhejiang, China; 2 Department of Infectious Diseases, Sir Run Run Shaw Hospital, Zhejiang University School of Medicine 26441 , Hangzhou, Zhejiang, China; 3 Department of Respiratory, Zhejiang Provincial People’s Hospital, People’s Hospital of Hangzhou Medical College, Hangzhou, Zhejiang, China; 4 Key Laboratory of Microbial Technology and Bioinformatics of Zhejiang Province, Hangzhou, China; 5 Regional Medical Center for National Institute of Respiratory Diseases, Sir Run Run Shaw Hospital, Zhejiang University School of Medicine 26441 , Hangzhou, Zhejiang, China; 6 Department of Pediatrics, Jinhua Women’s and Children’s Hospital, Jinhua, China; 7 Department of Infection Management, Affiliated Jinhua Hospital, Zhejiang University School of Medicine 26441 , Jinhua, Zhejiang, China; 8 Department of General Practice, Sir Run Run Shaw Hospital, School of Medicine, Zhejiang University 12377, Hangzhou, Zhejiang, China; Universita degli studi di roma La Sapienza, Rome, Italy

**Keywords:** *Pseudomonas aeruginosa*, ST463, IncP-2 megaplasmid, KPC-2, Tn*1403*-like transposon

## Abstract

The extensively drug-resistant (XDR) *Pseudomonas aeruginosa* ST463 strains, which co-harbor plasmid-associated metallo-β-lactamase (MBL) and *bla*_KPC-2_ genes, exhibit significant resistance and virulence, posing great clinical treatment challenges. Here, we report on three XDR *P. aeruginosa* ST463 strains, PA64, PA3117, and PA30, all carrying two plasmid types. One plasmid was a ~450 kb IncP-2-type megaplasmid named pPA64_1, pPA3117_1, and pPA30_1 in strains PA64, PA3117, and PA30, respectively. The other plasmid was a type I plasmid named pPA64_2, pPA3117_2, and pPA30_2 in strains PA64, PA3117, and PA30, respectively, harboring the *bla*_KPC-2_ gene in the core genetic platform IS*Kpn27- bla*_KPC-2_-IS*Kpn6*. The *bla*_KPC-2_ gene copies were associated with IS*26*-mediated inversion or duplication events. Notably, the IncP-2 megaplasmids pPA64_1, pPA3117_1, and pPA30_1 were associated with a variable ~57.3 kb Tn*1403*-like transposon named Tn*6485g*, Tn*6485h*, and Tn*6485f*, respectively. Tn*6485g* carried the MBL gene *bla*_IMP-45,_ which was located in the class 1 integron In*786*, followed by an IS*CR1*-associated *armA* module and the IS*26*-composite transposon Tn*6309*. On this basis, other IS*CR1*-associated modules (IS*CR1-qnrVC6*, IS*CR1-bla*_PER-1_, and IS*CR1-bla*_AFM-1_) were inserted between In*786* derivatives and IS*CR1-armA*, resulting in a novel transposon, Tn6*485h*, carrying two MBL genes, *bla*_IMP-45_ and *bla*_AFM-1_. In contrast to Tn6*485h*, Tn*6485f* had another inserted copy of IS*CR1-qnrVC6*. We inferred that the evolution of the Tn*1403*-like transposon might be driven by the recruitment of IS*CR1*-associated antimicrobial resistance (AMR) modules under antibiotic pressure in a clinical setting.

## INTRODUCTION

*Pseudomonas aeruginosa* is an opportunistic pathogen responsible for a wide range of hospital-acquired infections. This pathogen is often associated with conditions, such as cystic fibrosis infections, ventilator-associated pneumonia, urinary tract infections, otitis externa, osteoarthritic infections, and bloodstream infections (1). Carbapenem antibiotics are typically the first-line treatment for multidrug-resistant *P. aeruginosa* infections. However, the rate of carbapenem-resistant *P. aeruginosa* (CRPA) infections has rapidly increased in recent years due to the excessive use of carbapenem antibiotics (2). A global multicenter study published in *The Lancet* demonstrated that there were approximately 38,100 deaths worldwide as a result of CRPA infections in 2019 (3). The rapid spread of CRPA has emerged as a significant public health concern, posing a major threat to public safety.

There are several reasons for carbapenem resistance in *P. aeruginosa*, with the acquisition of the carbapenemase gene being the most significant mechanism (4). Among the carbapenemase genes, metallo-β-lactamase (MBL) genes, in particular *bla*_IMP_ and *bla*_VIM_, are the most common type in *P. aeruginosa* worldwide (5, 6). In 2018, a novel MBL gene, *bla*_AFM-1_, was first discovered in *Alcaligenes faecalis* in China (7) and subsequently identified in *P. aeruginosa strains* (8). ST463, initially identified as a predominant clone in east China, has emerged as a new potential high-risk clone due to its high virulence (9). Notably, ST463 is the only clone positive for both the *exoU* and *exoS* genes in *P. aeruginosa* (10). Moreover, the mortality rate associated with bloodstream infections caused by ST463 CRPA is higher than that associated with other clones (11). A significant proportion of ST463 strains carrying the carbapenemase gene *bla*_KPC-2_ is resistant to all β-lactam antibiotics, except ceftazidime/avibactam (12).

In this study, we isolated three strains of ST463 CRPA harboring both the *bla*_KPC-2_ and MBL genes. Of these strains, two carried *bla*_KPC-2_, *bla*_IMP-45_, and *bla*_AFM-1_ (PA30 and PA3117), whereas the remaining strain carried *bla*_KPC-2_ and *bla*_IMP-45_ (PA64). Notably, all carbapenemase genes were located on plasmids. We conducted a thorough analysis of these strains’ genetic characteristics and assessed the plasmids’ evolutionary patterns.

## MATERIALS AND METHODS

### Bacterial strain and antimicrobial susceptibility testing

Three *P. aeruginosa* strains were isolated from samples from inpatients at the Affiliated Jinhua Hospital, Zhejiang University School of Medicine in Zhejiang Province, China. Matrix-assisted laser desorption/ionization time-of-flight mass spectrometry (MALDI-TOF MS) was used to identify the strains. The antimicrobial susceptibility of the strains was determined via the broth microdilution method, and the results were interpreted according to the guidelines provided by the Clinical and Laboratory Standards Institute (CLSI) of 2024.

### Whole-genome sequencing and plasmid analysis

The genomic DNA of the three *P. aeruginosa* strains was extracted using the PureLink Genomic DNA Mini Kit (Invitrogen, Carlsbad, CA, USA) and subsequently sequenced on the Illumina HiSeq X10 (San Diego, CA, USA) and Nanopore MinION (Oxford, UK) platforms. The Illumina and Nanopore reads were then hybrid-assembled via Unicycler v0.4.8 (13). The resulting contigs were annotated using RAST (14). Multilocus sequence typing (MLST) was performed using the PubMLST database for *P. aeruginosa* (https://pubmlst.org/organisms/pseudomonas-aeruginosa). Resistance and virulence genes were identified via ABRicate 1.0.0 (https://github.com/tseemann/abricate). Plasmid sequence alignment was conducted via BLAST Ring Image Generator (BRIG) (15). Transposon Registry (16) and ISﬁnder (17) were utilized for identifying the transposons and insertion sequences, respectively. The further confirmations of the construction of the transposons were manually refined by BLASTn/BLASTp (18). Equal comparisons of the plasmids and transposons were performed using Easyfig (19).

### Conjugation experiments

Conjugation experiments were conducted using PA30, PA3117, and PA64 as the donor strains and a rifampin-resistant mutant of *P. aeruginosa* PAO1 (PAO1-RifR) as the recipient strain. Transconjugants were selected on Mueller-Hinton (MH) agar media supplemented with 800 µg/mL rifampin and 32 µg/mL ceftazidime. The conjugation experiments for each strain were repeated more than three times. The growing colonies on the selective plates were conﬁrmed via polymerase chain reaction (PCR) ampliﬁcation.

## RESULTS

### Clinical and microbiological characteristics

The PA30 strain was isolated from the catheter of a 67-year-old male patient who had undergone a clearance operation for an intracranial hematoma. The patient had a fever of 37.8°C after the operation and recovered after consecutive treatment with cephalosporins, linezolid, and meropenem. It was considered that the isolated PA30 strain was more likely related to colonization than to infection. The PA3117 strain was isolated from the bile drainage fluid of a 62-year-old female patient admitted to the Department of Hepatobiliary and Pancreatic Surgery. The patient was diagnosed with cholangitis after undergoing choledochotomy and T-tube drainage. A combination of vancomycin with levofloxacin and vancomycin with imipenem was applied successively, and the CRPA strain PA3117 was cultured after these treatments. The patient was cured after polymyxin treatment and T-tube removal. The remaining strain, PA64, was isolated from the sputum sample of a 73-year-old male patient who was treated for left pulmonary adenocarcinoma. After left upper lobectomy and mediastinal lymph node dissection, the patient received successive anti-infection treatments with cefazolin, cefoperazone/sulbactam, imipenem, levofloxacin, and vancomycin. The patient was discharged after treatment.

All three strains were resistant to piperacillin, cefepime, ceftazidime, imipenem, meropenem, aztreonam, amikacin, gentamicin, levofloxacin, ciprofloxacin, and ceftazidime-avibactam. However, they were susceptible to colistin. All the strains demonstrated an extensively drug-resistant (XDR) phenotype. Antimicrobial susceptibility testing showed that PA30 and PA64 were susceptible to cefiderocol, while PA3117 demonstrated intermediate susceptibility. This finding provided novel insights for guiding antimicrobial treatment strategies against these three strains.

The PA64, PA3117, and PA30 strains harbored two kinds of plasmids. One plasmid type was identified as an IncP-2-type megaplasmid carrying genes encoding MBLs (pPA64_1, pPA3117_1, and pPA30_1), whereas the other type carried one or two copies of the *bla*_KPC-2_ gene (pPA64_2, pPA3117_2, and pPA30_2) (Table 1). The single-nucleotide polymorphism (SNP) difference between PA30 and PA3117 was 3 (SNP = 3), and that between PA30 and PA64 was 0 (SNP = 0). We constructed a phylogenetic tree based on the SNPs (Fig. S1). The phylogenetic tree clustered primarily into three major clades. PA30, PA64, and PA3117 clustered closely together within the predominant major clade.

**TABLE 1:** Clinical characteristics and MICs for *Pseudomonas aeruginosa* strains^*c*^

Strain	Date	Source	ST	Virulence gene	I/C	ICU^*a*^	MICs (μg/mL)													Plasmid characteristics		
							PIP	FEP	CAZ	IMP	MEM	AZT	AK	GM	LEV	CIP	CZA	COL	FDC	Plasmid	Size(bp)	Carbapenemase genes
PA30	2021-08-28	Catheter	463	exoU^+^ /exoS^+^	C	Yes^*b*^	256	1,024	1,024	128	>1,024	64	>1,024	>1,024	128	16	>1,024/4	0.5	4	pPA30_1	453250	bla_IMP-45_, bla_AFM-1_
																				pPA30_2	49370	bla_KPC-2_, bla_KPC-2_
PA3117	2022-09-04	Bile	463	exoU^+^ /exoS^+^	I	Yes	>1,024	>1,024	>1,024	1,024	>1,024	>64	>1,024	>1,024	256	16	>1,024/4	0.5	8	pPA3117_1	449377	bla_IMP-45_, bla_AFM-1_
																				pPA3117_2	37887	bla_KPC-2_
PA64	2022-10-21	Sputum	463	exoU^+^ /exoS^+^	C	Yes	>1,024	>1,024	>1,024	1,024	>1,024	>64	>1,024	>1,024	256	16	>1,024/4	0.5	1	pPA64_1	426741	bla_IMP-45_
																				pPA64_2	37533	bla_KPC-2_
PA3117-PAO1-RifR	/	This study	/	/	/	/	512	>1,024	>1,024	64	>1,024	>64	>1,024	>1,024	2	1	>1,024/4	0.5	8	/	/	/
PAO1-RifR	/	This study	/	/	/	/	4	4	4	1	0.5	8	<0.5	2	<0.5	0.12	2/4	0.5	0.5	/	/	/
PAO1	/	This study	547	exoU^−^ /exoS^+^	/	/	4	4	2	1	1	4	<0.5	2	<0.5	0.06	1/4	1	0.5	/	/	/

^*a*^ ICU, intensive care unit.

^*b*^ Yes, the isolation of strain was before admission to the ICU.

^*c*^ I, infection; C, colonization;PIP, piperacillin; FEP, cefepime; CAZ, ceftazidime; IMP, imipenem; MEM, meropenem; AZT, aztreonam; AK, amikacin; GM, gentamicin; LEV, levofloxacin; CIP, ciprofloxacin; CZA, ceftazidime-avibactam; COL, colistin; FDC, cefiderocol./:not applicable.

We performed conjugation assays to examine the transferability of the megaplasmids. A transconjugant was obtained from the PA3117-PAO1-RifR conjugation experiment with a very low conjugation frequency (2.78 × 10^−14^). We failed to obtain transconjugants from PA64-PAO1-RifR and PA30-PAO1-RifR in more than three independent conjugation experiments. To further characterize the transconjugant, we conducted antibiotic susceptibility assays of the transconjugant PA3117-PAO1-RifR (Table 1). The results revealed that the transconjugant PA3117-PAO1-RifR maintained resistance profiles comparable to the donor strain PA3117 across all tested antibiotic classes, except for quinolones, to which it showed susceptibility.

### Genetic features of the plasmids

**Fig 1:**
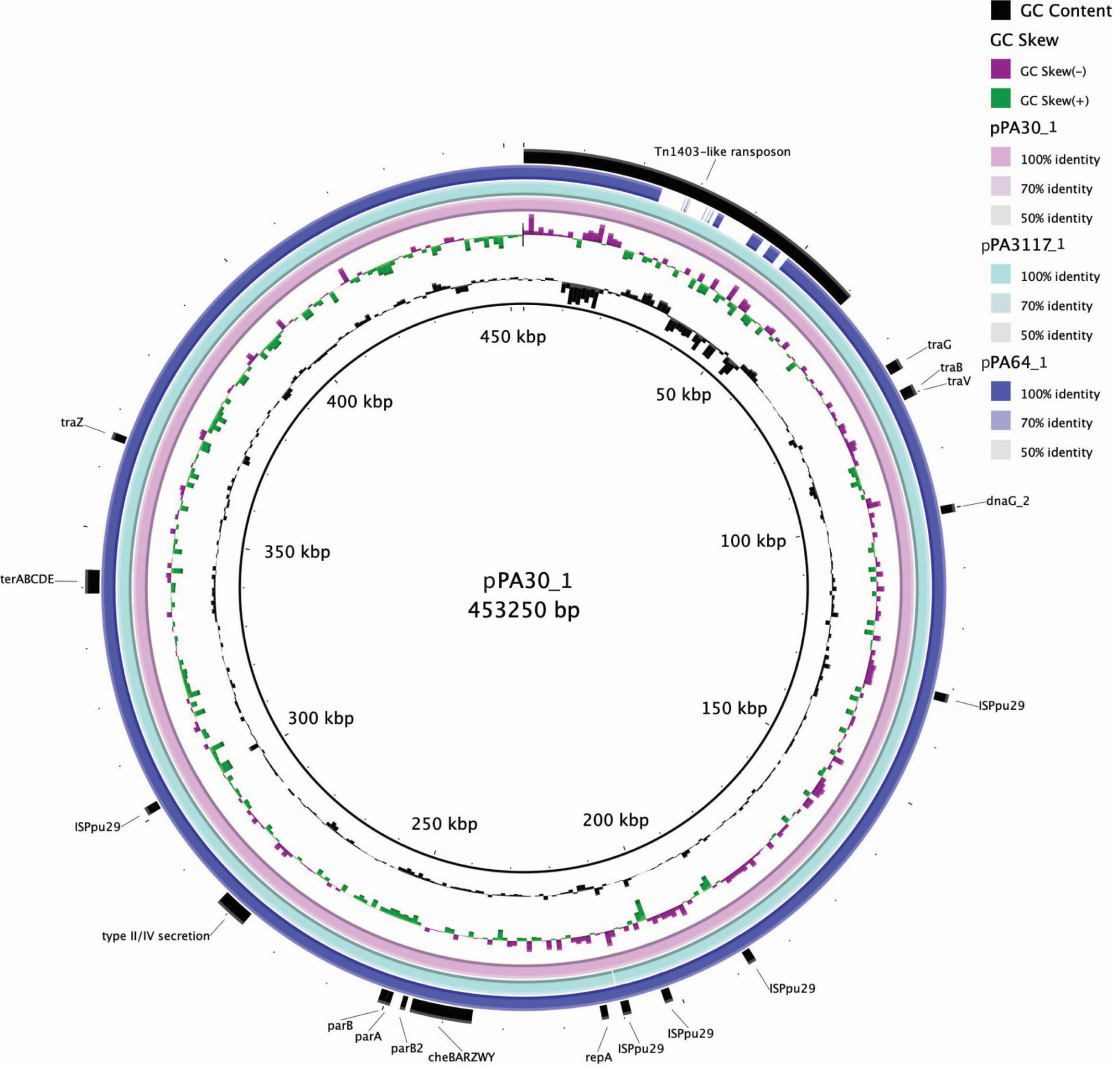
Genetic comparison of three IncP-2 megaplasmids. The sequence of pPA30_1 is taken as the reference. The innermost circle indicates the scale, and the second and third circles illustrate the GC content and skew, respectively. The colored circles from the inner to the outer represent each plasmid, as shown in the right column. The solid regions demonstrate a sequence similar to that of the reference, whereas the gaps represent regions lacking sequence similarity. The annotations on the outermost circle indicate the locations of the main features of pPA30_1.

The length of the base pairs of megaplasmids pPA64_1, pPA3117_1, and pPA30_1 was greater than 450 kb, and the plasmid backbones were highly similar, with over 95% query coverage and 99% nucleotide similarity. All three megaplasmids encoded identical RepA replication and ParAB partition proteins with 100% amino acid sequence identities. The genetic elements of the backbones were conserved and included the conjugative module *traGBV*, which is responsible for horizontal plasmid transmission. The tellurite resistance operon *terABCDEZ* and the chemotaxis operon *cheBARZW*Y, the intrinsic structures of IncP-2 megaplasmids, were also identified (Fig. 1).

**Fig 2:**
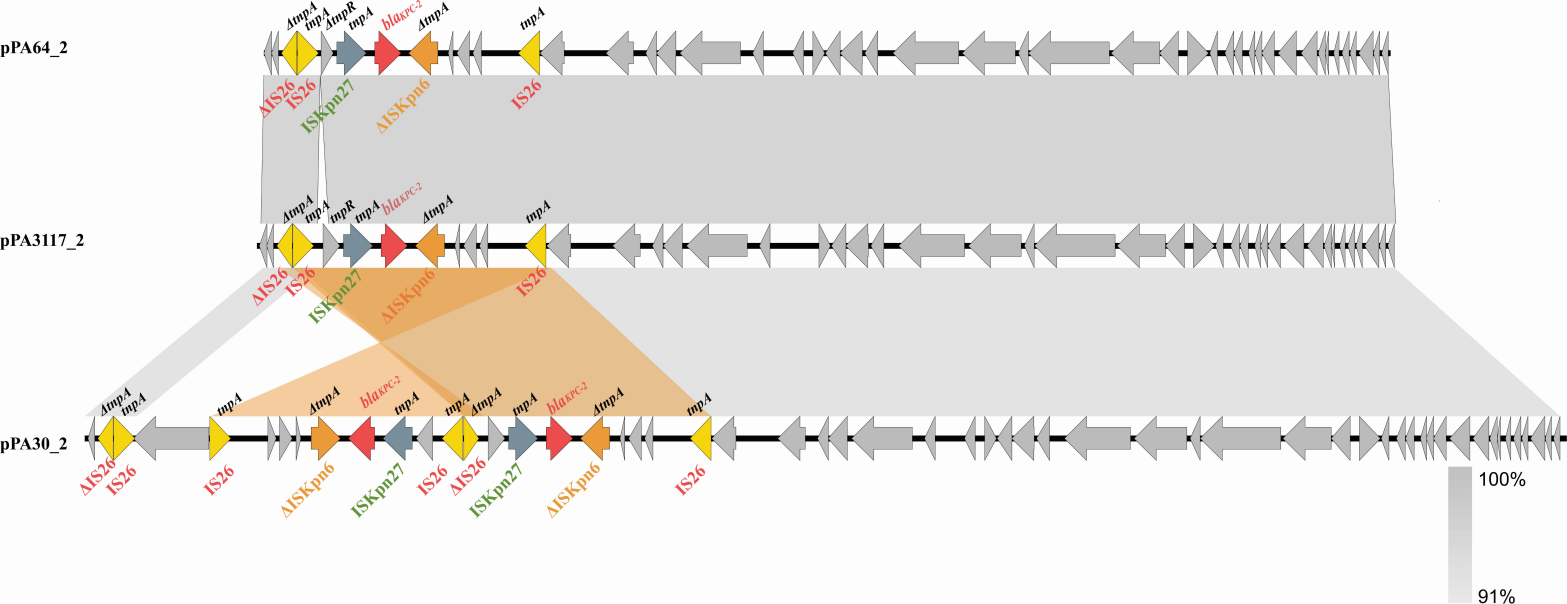
Alignment of the genetic context of pPA64_2, pPA3117_2, and pPA30_2. The shaded regions denote nucleotide identity (100%). The antibiotic resistance genes *bla*_KPC-2_ are denoted by red arrows. The IS elements IS*26*, IS*Kpn27*, and ΔIS*Kpn6* are denoted by yellow, blue, and orange arrows, respectively.

The complete plasmid sequences of pPA64_2 and pPA3117_2 were 37,533 and 37,887 bp long, respectively. These two plasmids shared identical backbones and carried a single copy of *bla*_KPC-2_ on the IS*Kpn27*-ΔIS*Kpn6* gene platform ﬂanked by two IS*26* insertion elements. The IS*26*-associated module was duplicated and inverted in pPA30_2, forming two symmetric IS*26-bla*_KPC-2_-IS*26* units and yielding two copies of the *bla*_KPC-2_ genes and adjacent IS*26* mosaic structures (Fig. 2).

### Tn*1403*-like transposon evolution in IncP-2 megaplasmids

The primary distinguishing regions among pPA64_1, pPA3117_1, and pPA30_1 were recognized as the Tn*1403*-like transposon regions carrying the antimicrobial resistance (AMR) genes encoding MBLs. A Tn*1403*-like transposon possessed *tnpA* and *tnpR,* demonstrating 99% amino acid sequence identity to those of Tn*1403* (20). The transposon Tn*1403* was an important vehicle for resistance gene dissemination, which featured a backbone structure organized as *tnpAR-res* site-*sup-uspA-dksA-yjiK* (7). Tn*6485*-like transposons, the derivatives of Tn*1403*, inserted *bla*_IMP-45_-bearing In*786* into the *res* site and retained the ancestral recombination module (*tnpAR-res* site) critical for transposition but lost most of the downstream Tn*1403* backbone elements, resulting in a truncated architecture specialized for disseminating the acquired antibiotic resistance genes (21). The megaplasmid pPA64_1 carried the MBL gene *bla*_IMP-45_ located in a novel Tn*6485*-like transposon named Tn*6485g*, which is closely related to Tn*6485b* from the plasmid pR31014-IMP (GenBank accession no. MF344571.1). The genetic architecture of the *bla*_IMP-45_ gene was identical in both Tn*6485*-like transposons, with an arrangement of antimicrobial resistance cassettes *aacA4-bla*_IMP-45_-*gcu*3-*bla*_OXA-1_-*catB3* in a class 1 integron In*786*. The 3′ conserved segment (3′CS) of the integron included the *qacE*Δ1 and *sul1* genes, followed by an identical array of genes, including the composition of the IS*CR1-armA* module, except for the IS*Ppu29* insertion element, which was absent in the IS*26*-composite transposon [IS*26-tetAR(C)-*IS*26*] in Tn*6485g* (Fig. 2).

**Fig 3:**
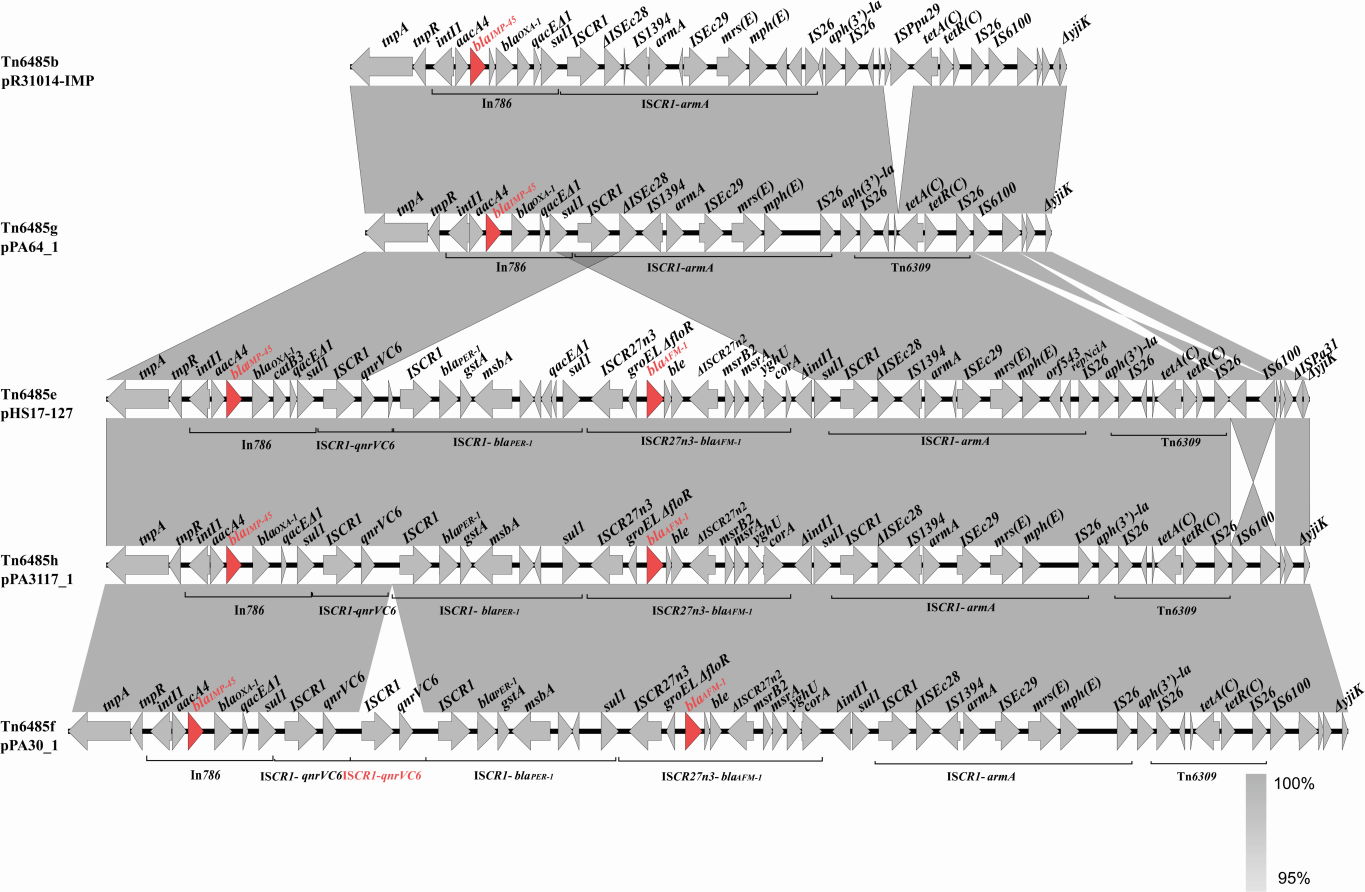
Alignment of the genetic context of Tn*1403*-like transposons from pPA64_1, pPA3117_1, and pPA30_1. The shaded regions denote nucleotide identity (95–100%). Red arrows denote the genes *bla*_IMP-45_ and *bla*_AFM-1_.

Compared to Tn*6485g* in pPA64_1, pPA3117_1 carried another novel Tn*6485*-like transposon named Tn*6485h*, which was very similar to Tn*6485e* in pHS17-127 (GenBank accession no. CP061377.1) and acquired an additional MBL gene, *bla*_AFM-1_, and the *bla*_PER-1_ and *qnrVC6* genes via the insertion of IS*CR* modules. The genetic environment of *bla*_AFM-1_ in pPA3117_1 was bracketed by two IS*CR27*-like elements (IS*CR27n3* and ΔIS*CR27n1*) in a conserved region of *groEL/*Δ*groEL-*Δ*ﬂoR-bla*_AFM-1_-*ble-*Δ*trpF-*ΔIS*CR27n2*-ΔIS*Pme1-msrB2-msrA-yghU-corA*. The AMR genes *bla*_PER-1_ and *qnrVC6* are related to the IS*CR1-qnrVC6* and the IS*CR1-bla*_PER-1_ modules located between In*786* and the IS*CR27n3-bla*_AFM-1_ module. In PA30_1, the transposon Tn*6485f* also harbored two MBL genes, *bla*_IMP-45_ and *bla*_AFM-1_, and one more IS*CR1-qnrVC6* module was identified upstream of the IS*CR1-bla*_PER-1_ module that was not present in Tn*6485h* (Fig. 3).

## DISCUSSION

ST463 has recently emerged as a novel potential high-risk clone in China because of its notable virulence and resistance traits. Previous studies have shown that 40.4% of CRPA carrying *bla*_KPC-2_ in China and ST463 represent the predominant clone in *bla*_KPC-2_-positive CRPA populations (12). The *bla*_KPC-2_ gene in the ST463 strains is carried mainly by type I plasmids. The core genetic platform harboring *bla*_KPC-2_ is IS*Kpn27- bla*_KPC-2_ -IS*Kpn6,* and the adjacent region varies by IS*26*-mediated inversion or duplication events, which amplify *bla*_KPC-2_ gene copies and facilitate the transmission of *bla*_KPC-2_ in ST463 strains (22).

Outbreaks of CRPA producing IMP-45 occurred in Shanghai, China in 2015, with the study identifying *bla*_IMP-45_ in 9.83% of CRPA strains (23). CRPA strains co-producing IMP-45 and AFM-1 have been documented in China (7). In these strains, both *bla*_IMP-45_ and *bla*_AFM-1_ genes reside on the same IncP-2-type plasmid within *P. aeruginosa*, and this co-harboring plasmid has been confirmed to be conjugative, facilitating horizontal gene transfer. Additionally, CRPA strains co-producing KPC-2 and AFM-1 (*bla*_KPC-2_ and *bla*_AFM-1_) have also been detected in China (24). Unlike the IMP-45/AFM-1 co-producers, in these isolates, *bla*_KPC-2_ was plasmid-borne, whereas *bla*_AFM-1_was chromosomally integrated. Epidemiological surveillance data highlighted the clinical significance: among 192 CRPA strains analyzed, eight (4.17%) co-harbored *bla*_KPC-2_ and *bla*_AFM-1_, and infections caused by these strains resulted in five patient fatalities (25).

In this study, we identified an ST463 *P. aeruginosa* strain (PA64) harboring both *bla*_KPC-2_ and *bla*_IMP-45_, the latter of which was carried by an IncP-2-type megaplasmid that is highly related to the spread of MBL genes in *P. aeruginosa* (23, 26). The IncP-2 megaplasmids share a common core genetic backbone that includes genes involved in replication, segregation, and conjugation, improving the efficiency of vertical and horizontal transmission of megaplasmids (27). These beneficial traits of megaplasmids provide adaptive advantages for acquiring and disseminating resistance genes. The extensively variable regions are associated with multiple cassette-borne AMR genes, such as *bla*_VIM-2_ genes in In*461* and *bla*_IMP-45_ genes in the variable region of In*786*, encoding MBLs disseminated in nosocomial *P. aeruginosa* isolates (23, 26). The *bla*_IMP-45_ gene in PA64 was also carried in In*786* located in an ~57.3 kb Tn*1403*-like transposon newly named as Tn*6485g*. Tn*1403* was recovered from a multiple antibiotic-resistant clinical strain of *P. aeruginosa* and acted as a vehicle for a series of resistance modules associated with MGEs (20). The Tn*1403* backbone contains the *tnpA* and *tnpR* genes, which encode the TnpA transposase, TnpR resolvase, and most of the *res* site. The *bla*_IMP-45_-bearing In*786* derivatives were embedded within the *res* site but lost most of the Tn*1403* backbone structure, followed by an IS*CR1*-associated *armA* module and an IS*26*-composite transposon Tn*6309* (7).

On this basis, another novel Tn*1403*-like transposon, Tn6*485h*, which was inserted by various IS*CR1*-associated modules (IS*CR1-qnrVC6*, IS*CR1-bla*_PER-1_, and IS*CR1-bla*_AFM-1_), was identified from the IncP-2 megaplasmid of the ST463 *P. aeruginosa* strain PA3117. Additionally, one more copy of IS*CR1-qnrVC6* was inserted, resulting in Tn*6485f* of pPA30_1. This finding suggests that the Tn*1403*-like transposons are highly variable in terms of the recruitment of IS*CR1*-associated modules carrying multiple AMR genes, which are captured by the IncP-2 megaplasmid and exhibit multiple drug resistance (MDR) properties in the host. The evolution of these MDR transposons may be driven by the pressure of antibiotic therapy in a specific clinical setting since all three patients with infections with novel Tn*1403*-like transposons in our study were admitted to the same ICU ward. The small SNP differences between these three isolates indicated that they were genetically indistinguishable at the core genome level and likely represented a recent clonal transmission event within the healthcare setting. This finding provided strong molecular evidence for nosocomial cross-transmission and underscored the need for targeted infection control interventions. However, the details of the evolutionary path are lacking, and the realities are likely more complex than anticipated.

In conclusion, we report on three clinical XDR *P. aeruginosa* ST463 strains, all containing two plasmids. One plasmid is an IncP-2 megaplasmid containing a variable Tn*1403*-like transposon that might be modified by recruiting IS*CR1*-associated AMR modules under antibiotic pressure in a clinical setting. The other plasmid, which is common in ST463 strains, harbors the *bla*_KPC-2_ gene in the core genetic platform IS*Kpn27- bla*_KPC-2_- IS*Kpn6*, and the *bla*_KPC-2_ gene copies are associated with IS*26*-mediated inversion or duplication events. These transferable IncP-2 megaplasmids, which harbor XDR transposons and coexist with plasmids carrying *bla*_KPC-2_ genes in *P. aeruginosa* ST463 strains, pose a substantial challenge to clinical treatment.

## Data Availability

The data used in our research are available for access. The complete sequences of the chromosomes and plasmids of PA64 and PA3117 have been submitted to GenBank under the BioProject accession numbers PRJNA1123618 and PRJNA224116, respectively. The BioProject accession number of PA30 is PRJNA866310.

## SUPPLEMENTAL MATERIAL

Fig. S1 aac.01697-24-s0001.docx Phylogenetic tree of the ST463 strains isolated in China from the NCBI Pathogen Database and the three strains (PA30, PA64, and PA3117) from this study.

ASM does not own the copyrights to Supplemental Material that may be linked to, or accessed through, an article. The authors have granted ASM a non-exclusive, world-wide license to publish the Supplemental Material files. Please contact the corresponding author directly for reuse.
