# Supplementary material for: Evolution of XDR Pseudomonas aeruginosa ST463 strains with two plasmids harboring multiple antimicrobial resistance genes
Source: Antimicrob Agents Chemother. 2025 Jul 23;69(9):e01697-24. doi: 10.1128/aac.01697-24 (PMC12406656; doi:10.1128/aac.01697-24)

**Supplementary figure. The phylogenetic tree of ST463 strains isolated in China from the NCBI Pathogen Database and the three strains (PA30, PA64, and PA3117) from this study.** All strains without definitive isolation time and location information were removed during the screening process. Snippy-multi was utilized for calculating the single nucleotide polymorphism (SNP) differences and FastTree was used to construct a phylogenetic tree. The midpoint rooting method in iTOL v6.1.1 was used to distinguish the groups.


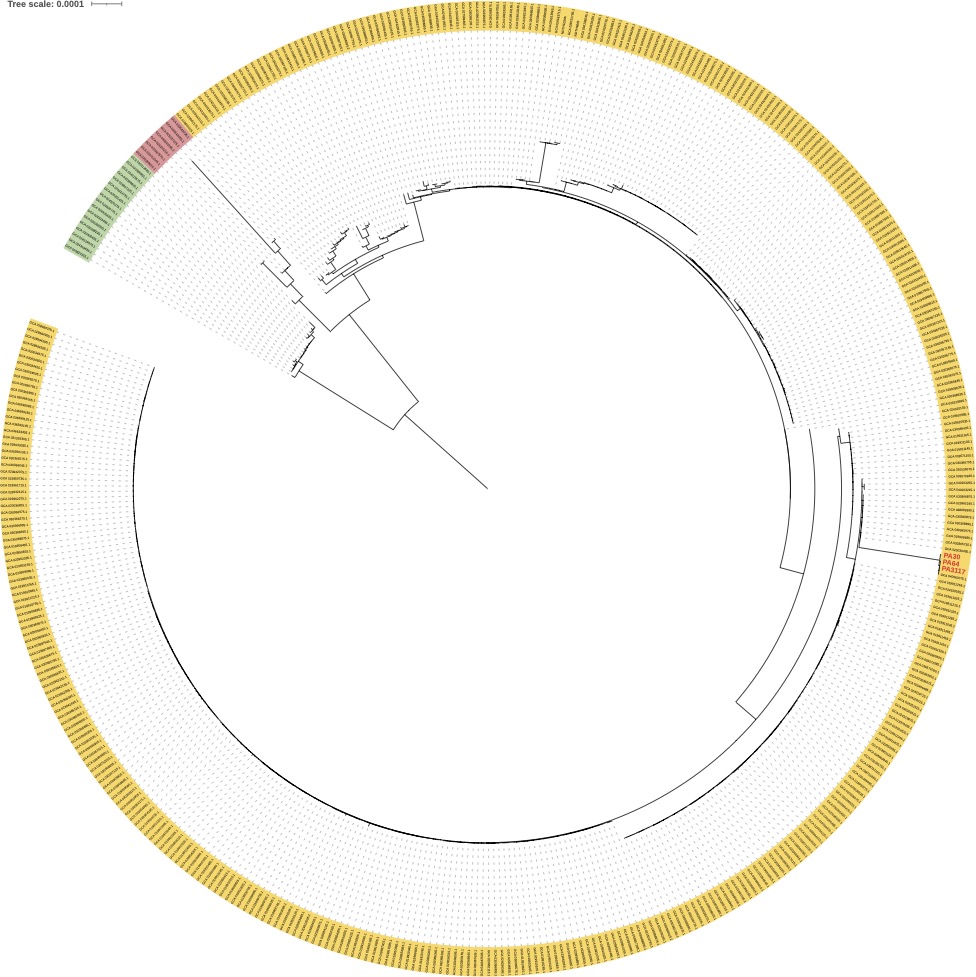

Supplement: Fig. S1 — Phylogenetic tree of the ST463 strains isolated in China from the NCBI Pathogen Database and the three strains (PA30, PA64, and PA3117) from this study. [file aac.01697-24-s0001.docx]
